# Food for the Mind: A systematic review of mindful and intuitive eating approaches for mental health & wellbeing

**DOI:** 10.1192/j.eurpsy.2024.352

**Published:** 2024-08-27

**Authors:** M. Eaton, T. Foster, J. Messore, L. Robinson, Y. Probst

**Affiliations:** ^1^School of Medical, Indigenous and Health Sciences; ^2^School of Psychology, University of Wollongong, Wollongong, Australia

## Abstract

**Introduction:**

A growing body of literature has investigated diet and mental health, however, it is often viewed through a “weight-centric” lens, where weight loss is considered a primary outcome and motivator. This review aims to shed new insights into the connections between mental health and wellbeing, and eating behaviours that focus on internal cues and regulators and do not centralise around weight. Such “weight-neutral approaches” have been associated with improved psychological health and wellbeing, however, consolidated evidence is lacking.

**Objectives:**

To explore eating styles that do not centralise around weight, and their relationship with mental health and wellbeing and other health outcomes.

**Methods:**

A systematic search was performed including observational studies of adult populations, with ≥1 mental health and wellbeing or physical health outcome, and ≥1 validated measure of eating behaviour reflective of a weight-neutral approach. Outcomes were characterised into four domains (mental health and wellbeing, physical health, health promoting behaviours and other eating behaviours). Risk of bias was assessed using the Newcastle-Ottawa Scale.

**Results:**

In total 8281 records were identified with 86 studies including 75 unique datasets and 78 unique exposures included. Eating behaviours included intuitive eating (n=48), mindful eating (n=19), and eating competence (n=11). All eating behaviours incorporated biological, physiological, and social factors, with 297 outcomes categorised for mental health and wellbeing (n=122), physical health (n=116), health promoting behaviours (n=51) and other eating behaviour (n=8). Greater intuitive and mindful eating were significantly related to lower levels of disordered eating, and depressive symptoms, as well as greater body image, self-compassion, and mindfulness. Greater intuitive eating, mindful eating and eating competence were significantly related to a lower BMI, and greater diet quality and physical activity. Eating competence and intuitive eating were significantly related to higher fruit and vegetable intake, and eating competence alone was significantly related to higher fibre intake, and greater sleep quality.

**Conclusions:**

This review provides evidence that intuitive eating, mindful eating and eating competence are positively related to a range of mental and physical health outcomes. Considered within the biopsychosocial model, these findings enhance understanding around the impact of approaches to healthy eating patterns that are not focused on weight loss, and contributes a case towards promoting health-centric eating behaviour in mental health care. Future research should focus on experimental studies and broader population groups.

**Disclosure of Interest:**

None Declared

